# Layer Embedding Analysis in Convolutional Neural Networks for Improved Probability Calibration and Classification

**DOI:** 10.1109/TMI.2020.2990625

**Published:** 2020-10-28

**Authors:** Fan Zhang, Nicha Dvornek, Junlin Yang, Julius Chapiro, James Duncan

**Affiliations:** Department of Biomedical Engineering, Yale University, New Haven, CT, 06511, USA; Department of Radiology and Biomedical Imaging, Yale University, New Haven, CT, 06511, USA; Department of Biomedical Engineering, Yale University, New Haven, CT, 06511, USA; Department of Radiology and Biomedical Imaging, Yale University, New Haven, CT, 06511, USA; Department of Biomedical Engineering, Yale University, New Haven, CT, 06511, USA

**Keywords:** layer embeddings, network interpretability, probability calibration, convolutional neural networks, liver tissue classification

## Abstract

In this project, our goal is to develop a method for interpreting how a neural network makes layer-by-layer embedded decisions when trained for a classification task, and also to use this insight for improving the model performance. To do this, we first approximate the distribution of the image representations in these embeddings using random forest models, the output of which, termed embedding outputs, are used for measuring how the network classifies each sample. Next, we design a pipeline to use this layer embedding output to calibrate the original model output for improved probability calibration and classification. We apply this two-steps method in a fully convolutional neural network trained for a liver tissue classification task on our institutional dataset that contains 20 3D multi-parameter MR images for patients with hepatocellular carcinoma, as well as on a public dataset with 131 3D CT images. The results show that our method is not only able to provide visualizations that are easy to interpret, but that the embedded decision-based information is also useful for improving model performance in terms of probability calibration and classification, achieving the best performance compared to other baseline methods. Moreover, this method is computationally efficient, easy to implement, and robust to hyper-parameters.

## I. Introduction and Background

HEPATOCELLULAR carcinoma (HCC) is one of the most common malignancies in the liver, accounting for approximately 75 percent of all liver cancers, and is the third most common cause of cancer-related death worldwide [1]. Upon diagnosis, it is frequently treated with image-guided minimally invasive therapies that can deliver treatments directly to the tumor while leaving the remaining liver tissue unharmed. However, such therapies, including the catheter-based transarterial chemoembolization (TACE), are known to have variable efficacy depending on tumor characteristics [2]. Advanced imaging, such as multi-parameter dynamic contrast enhanced (DCE) magnetic resonance (MR) imaging, is useful in quantifying such characteristics, evaluating a patient’s amenability to such therapies, and estimating tumor response [3].

**Fig. 1.**
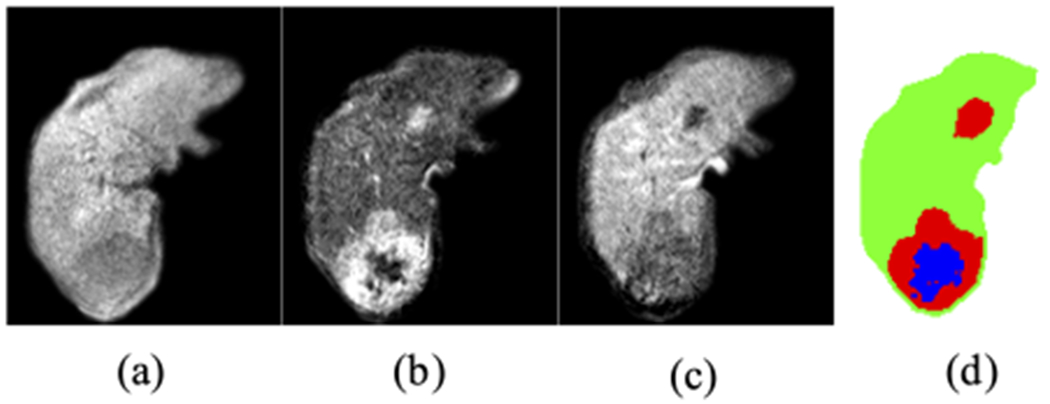
Demonstration of multi-parameter T1-weighted dynamic contrast enhanced MR images and an expert-delineated tissue classification map. (a) pre-contrast; (b) arterial phase; (c) venous phase; (d) tissue classification ground truth, where green represents healthy liver tissue, red viable tumor tissue, blue necrotic tissue.

Therefore, in our application, we first are interested in developing an algorithm that can automatically classify the different tissue types within the liver from 3D multi-parameter MR images, including healthy liver tissue and anomalies, which consist of viable tumor tissue and necrosis (Fig. 1). Such tissue quantification can serve for both evaluating treatment outcome and identifying biomarkers for predicting patients’ overall survival rate [4]. However, manual labelling of a 3D image on tissue classification is laborious and often subject to inter-rater variance. Thus, having a reliable automatic 3D segmentation algorithm is a most desirable goal.

Convolutional neural networks (CNN) have become the backbone of many vision recognition tasks, and their development has been rapid in recent years. These deep-learning-based models are able to extract image features automatically layer by layer to capture the inherent image data structure when given a large amount of well-curated data [5], [6]. However, such methodology is often criticized for its lack of interpretability on how a prediction is made through layers of non-linear transformations. This “black-box” like characteristic is particularly undesirable when such models are applied in the medical domains [7] where the reliance of the model on clinically understandable and reliable/reproducible image-derived information is paramount. Thus, the interpretability discussed here is beneficial from two perspectives. From the users’ perspective, the model’s reasoning behind its outputs can be understood intuitively, where this is also defined as explainability. From the developers’ perspective, such insight into how a model is making decisions can be useful for improving model performance. In this work, we are focusing on the latter perspective.

Compounding the difficulties with interpreting outputs of decision-making neural network architectures/models is the need to assign a measure of certainty to the estimated results. We call this “probability calibration” regarding the estimated output. In a classification task, the model should output a reliable probability distribution on all potential classes, properly reflecting the model’s certainty in its prediction. However, for most neural network architectures, such a probability estimation is often absent or highly inaccurate (e.g. a 95% score actually only has 20% likelihood of being correct). This introduces the need to *calibrate* the model output, which is the process of adjusting the model output to have a true probabilistic meaning, thus improving its probability calibration. We review work in the two areas of neural network interpretability and probability calibration in the following subsections.

### a) Previous work on interpretation of neural network:

There has been a large variety of effort on neural network interpretation. Here, we briefly overview some major directions, and the most related ones will be introduced in more detail in the corresponding method sections.

- *Channel Feature Interpretation* methods aim to look at the meaning of a certain channel in a neural network. Among them, activation maximization methods [8], [9] look to find image input patterns that can maximize the activation value of a certain channel. On the other hand, layer-wise relevance propagation [10] methods derives a similar outcome by running a backpropagation in the network, redistributing the output score to each layer channel. Both of these methods can qualitatively inform the user about the types of input patterns that the neural network is looking for when making a decision.
- *Input Inversion* methods seek to visualize what information is preserved in a certain layer. Since a neural network is expected to concentrate on the portion of information of the input image that is important for the task, and ignore the irrelevant part, such visualization can be helpful in model decision interpretation. Among them, patch correspondence [11], [12] methods create such visualizations by directly looking for similar image samples from the training set that generate a similar activation. Others, such as inversion [13]–[15] methods, find such visualizations through various ways of backward optimization.
- *Mutual Information* methods is one of the more quantitative methods for analyzing a neural network model. In particular, the information bottleneck [16] work is successful in computing the mutual information between each layer and input/output in some simple dataset, revealing intriguing model dynamics during the training and convergence.

However, we find that most of these efforts often present a result that is solely for the purpose of interpretation, and are not used to impact the original task. In other words, once the model gives its prediction output regarding a certain input, and the interpretation algorithm finishes rendering a visualization or quantification for interpreting the model, there is no further deliberation in terms of how we can use these interpretation results to improve the model performance. In this work, our intuition is that the insight into the model should help probability calibration, especially if we know how the model is making decisions layer by layer.

### b) Previous efforts on probability calibration of neural network outputs:

The most notable work on probability calibration uses a simple linear scaling function for adjusting the output magnitude [17], [18]. One could also calibrate the output using a piece-wise constant function [19] or advanced histograms binning techniques [20]. On the other hand, enforcing a well calibrated probability output can also be used as a way to regularize the neural network [21], improve robustness against adversarial attacks [22], or detect out-of-distribution examples [23]. Parallel to the works in probability calibration is a line of effort on neural network uncertainty estimation. The two concepts are closely related, and the latter direction has also inspired us in our own study. It was first proposed that one could use Bayesian theory to estimate the distribution of weights in the neural network for model uncertainty quantification [24]–[26]. Later, techniques were developed to approximate such model variation with a lower computational cost, such as Monte Carlo dropout [27], [28], ensemble method [29], and test-time augmentation [30]. Generally, these methods also improve the robustness and generalizability of the neural network model. In our experience, as well as reported in the literature [31], we find that such uncertainty estimation algorithm can also be used to improve probability calibration. Among all of these works, to the best of our knowledge, we did not find one that utilizes the decision making mechanism of the trained model itself to help with probability calibration.

Therefore, in this study, we are interested in the following three aims:

1. We want to establish a baseline segmentation model for liver tissue classification on MR images using a convolutional neural network.
2. We aim to analyze how the network casts input images into different representations at each of its layers embeddings progressively, in order to make a prediction.
3. We hope to use this insight of layer embeddings to improve the performance of the model in terms of probability calibration and classification.

To accomplish this, we adopt a minimal fully convolutional network (FCN) architecture that is not only able to provide a reasonable segmentation performance, but is also easy for the subsequent model interpretation and improvement. During the layer embedding analysis step, we construct statistical models to approximate the data distribution in each layer’s embedding. The outputs of such statistical models, termed layer embedding outputs, provide us a way to quantitatively estimate how the network is arranging different classes of data across layers to reach the final decision.

Furthermore, to improve the overall model performance in terms of probability calibration and classification, we create a pipeline that uses the layer embedding output to calibrate the original output. Such a prototype is based on our intuition that the network should solve easier tasks in earlier layers and difficult ones later. Through a comparison with several baselines on our institutional dataset as well as a public dataset, we demonstrate that our layer embedding analysis method is helpful in analyzing and improving the FCN model and has potential for investigating other types of neural networks.

### A. Contributions

The contribution of our work is summarized as follows:

- We design an innovative way of investigating the embeddings in each layer of the FCN, providing both quantitative measurements and intuitive visualizations. It is efficient to compute and robust to hyper-parameter tuning by utilizing random forest models.
- To the best of our knowledge, this is the first work that is able to utilize the interpretation results for improving model performance in probability calibration and classification. It is accomplished by our design of a pipeline that calibrates the original FCN output using the intermediate layer embedding output.
- We design a novel way of decomposing the model into a series of the most basic operations, that, to the best of our knowledge, for the first time reveals the intriguing patterns of how the network reaches its final output quantitatively with a high level of details.

## II. Baseline Segmentation Network

**Fig. 2.**
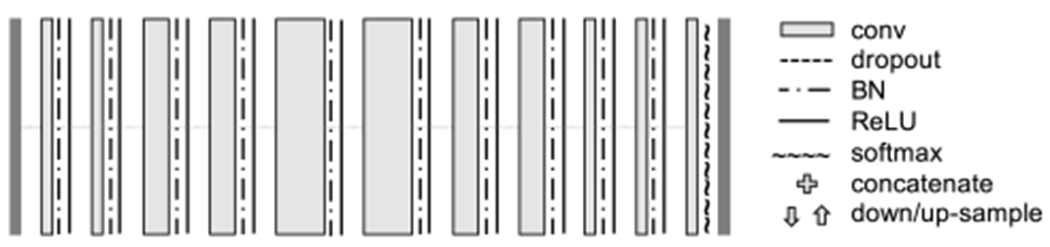
The fully convolutional network architecture adopted in our study and its legend.

To achieve the goal of tissue classification in 3D liver images, we initially adopt a fully convolutional neural network (FCN) design (Fig. 2). In this architecture, the whole network consists only of convolutional layers, batch normalization, and activation layers without any skip connections. We heavily utilize *dilated convolutions* to control the network receptive field in each layer. It is a type of convolution that introduces sparsity to its kernels by setting values at non-corner or non-center locations to zeros. Popularized by the works from DeepLab [32], it has the advantage of increasing the field-of-view in each convolution but keeping the number of parameters low. It is important to note that when we choose this minimal architecture of the neural network, our focus is to achieve a design that is convenient for the subsequent layer embedding analysis, and also has a reasonable (though at times can be flawed) classification performance. More specifically, the advantages of this architecture include:

- The implementation of dilated convolutions preserves the spatial dimension in each layer, which allows us to compare representations easily across different layers for the subsequent layer embedding analysis.
- Without the skip connections, the output value at each layer is strictly only a function of the output value from the previous layer. The signal processing inside the model thus can behave in a Markovian manner.
- Without max-pooling and max-unpooling layers, the model is able to accept a dynamic input size. We can train the model in patch-wise manner, and run predictions in test time using the whole image regardless of its size, introducing great efficiency for implementation.

## III. Layer Embedding Analysis

The ability for the convolutional neural networks to map the complex input image data into different embeddings layer by layer with respect to the end goal has been the underlying driving force for its outstanding performance in various computer vision tasks. Here, when we are talking about a *layer embedding*, we are referring to the space in which the representations from that hidden layer distribute.

One of the most popular ways of investigating data distribution in a high dimensional embedding is to use the t-SNE algorithm [33]. The goal of it is to create a low dimension point set where their pairwise distances mimic the ones in the original high dimensional space in a probabilistic sense. Though able to create intuitive visualizations, it is computationally demanding and not suitable for quantification.

One way to quantitatively measure how well the embedding separates different classes of samples is to model the data distribution in this embedding. To be specific, for the embedding in a certain layer *l*, assuming the training set image patches $\left\{I_{i}^{\left(\mathrm{train}\right)}\right\}$ with ground truth label $\left\{Y_{i}^{\left(\mathrm{train}\right)}\right\}$ are projected to representations $\left\{h_{i}^{\left(\mathrm{train},l\right)}\right\}$ in the neural network model *F*

(1) $$\left\{h_{i}^{\left(\mathrm{train},l\right)}\}=F^{\left(l\right)}(\{I_{j}^{\left(\mathrm{train}\right)}\}\right)$$

we can then construct another model, or equivalently a classifier, *M* for layer *l* using

(2) $$M^{\left(l\right)}=\underset{m}{\mathrm{argmin}}\|m(\{h_{i}^{\left(\mathrm{train},l\right)}\})-\{Y_{i}^{\left(\mathrm{train}\right)}\}\|$$

which outputs a probability distribution of potential labels for a sample point *I_j_* given its representation $h_{j}^{\left(l\right)}$ in the embedding

(3) $$\sigma_{j}^{\left(l\right)}=M^{\left(l\right)}(h_{j}^{\left(l\right)})$$

We call this probabilistic output *σ*^(*l*)^ the *embedding output*, which shows the relative location of the sample point in the embedding. If we have the ground truth *Y_j_* for *I_j_*, as is the case for the training set or validation set, we can also quantify the performance of the layer embedding output using metrics computed from $\sigma_{j}^{\left(l\right)}$ and *Y_j_*.

### A. Deep k-Nearest neighbor

The deep k-nearest neighbors (DkNN) technique [34] is one that uses a k-nearest neighbor model, one of the non-parametric methods, for constructing *M*’s. Originally designed to counter adversarial attacks [35], the idea behind this method is to investigate the k-nearest neighbors from the training set in the embedding space when a new data point is presented, similar to the philosophy used in the works on patch correspondence [12]. To be specific, for a test image patch $I_{j}^{\left(\mathrm{test}\right)}$, the goal is to find the set of k-nearest neighbors $\Omega_{\mathrm{kNN}}^{\left(l\right)}$ around $h_{j}^{\left(\mathrm{test}\right)}$ from $\left\{h_{i}^{\left(\mathrm{train},l\right)}\right\}$, and use its histogram to estimate $\sigma_{j}^{\left(l\right)}$.

(4) $$\Omega_{\mathrm{kNN}}^{\left(l\right)}=\text{k}\;\underset{i}{\mathrm{argmin}}\|h_{j}^{\left(\mathrm{test},l\right)}-h_{j}^{\left(\mathrm{train},l\right)}\|\;\sigma_{j}^{\left(l\right)}=\mathrm{hist}({\left\{Y_{i^{\prime }}^{\left(\mathrm{train}\right)}\right\}}^{\left(l\right)},\;i^{\prime }\in \Omega_{\mathrm{kNN}}^{\left(l\right)})$$

However, the high computational cost of the kNN search renders this algorithm inapplicable when the training set size $\left|\{I_{i}^{\left(\mathrm{train}\right)}\}\right|$ is very large and when we also want to compute embedding output $\sigma_{j}^{\left(l\right)}$ for every image patch *j* in the test set image at every layer *l*. A more efficient way of estimating the embedding output is needed.

### B. Proposed Method: Deep Random Forest

**Fig. 3.**
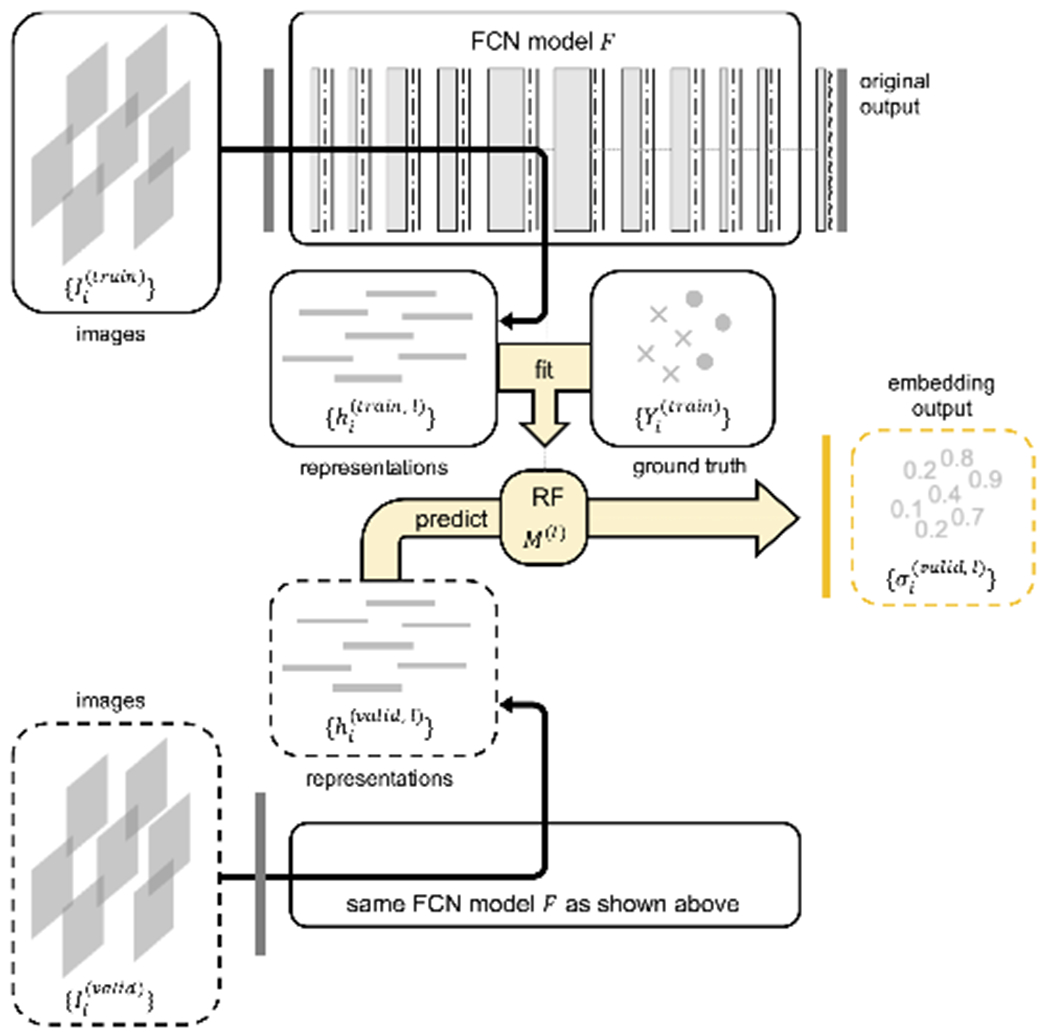
Deep random forest method for calculating layer embedding output. Solid line boxes represent training set data, dash line boxes unseen data (validation set or test set). The model at the bottom for the unseen data is the same model as the trained one at the top. Shapes in the ground truth box represent two different classes of labels. Random forest model ***M***^(*l*)^ is trained from representations ***h***^(*l*)^ and their ground truth labels ***Y*** in the training set to generate embedding output ***σ***^(*l*)^ = ***M***^(*l*)^ (***h***^(*l*)^) = ***M***^(*l*)^ (***F***^(*l*)^ (***I***))

Given representations $\left\{h_{i}^{\left(\mathrm{train},l\right)}\right\}$ and its ground truth labels $\left\{Y_{i}^{\left(\mathrm{train}\right)}\right\}$ from the training set in the embedding, instead of using a non-parametric estimator like kNN to approximate the local embedding space, we opt to use random forest [36] models to approximate the global embedding space at layer *l* (see Fig. 3). Compared to kNN, random forest is much more efficient computationally, especially when our training set size $\left\{h_{i}^{\left(\mathrm{train},l\right)}\right\}$ and test set size $\left|\{h_{i}^{\left(\mathrm{test},l\right)}\}\right|$ are both very large. Moreover, random forest is also known for its robustness to hyper-parameter settings.

To compute $\left\{\sigma_{j}^{\left(l\right)}\right\}$ for all test set image patches $\left\{I_{j}^{\left(\mathrm{test}\right)}\right\}$, we adopt the procedure shown in Algorithm 1. Note that we change the test set data to validation set because we want to keep the test data unseen when analyzing the layer embeddings, in preparation for the next aim of our study, which is to ultimately use this embedding output to improve model performance (section IV).

**Algorithm 1:** Deep random forest: layer-wise embedding analysis using random forest models.

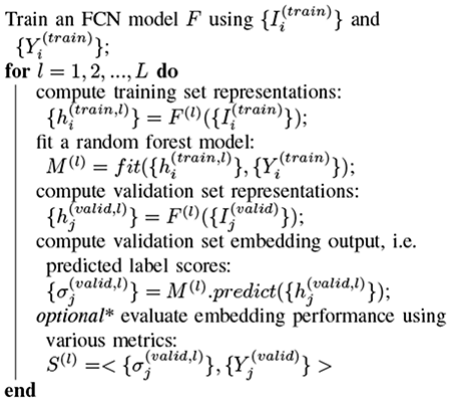

### C. A Breakdown of FCN Model

**Fig. 4.**
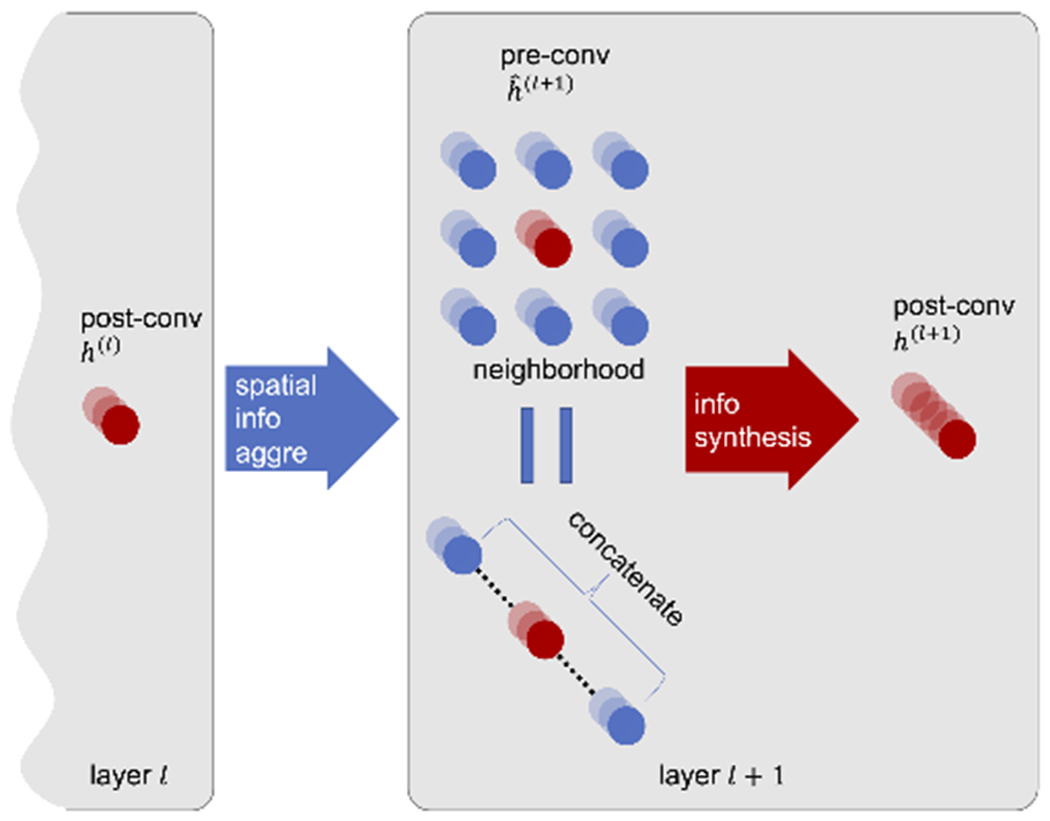
Convolution layer breakdown into spatial information aggregation (blue arrow) and information synthesis (red arrow). Red nodes symbolize the representation vector at a certain voxel location, and blue nodes symbolize the representation vectors in the neighborhood of that voxel.

To have a more microscopic view of how the embeddings evolve, we break down the process of how the representations change from one layer to the next into a two step process (Fig. 4): a *spatial information aggregation*, where all the neighboring voxel information is aggregated together, being equivalent to a concatenation, and *information synthesis*, where all the neighborhood information is compressed by the dilated convolution, batch normalization, and activation function, keeping some neighborhood information and discarding others. In general, by having this breakdown, we are interested in seeing how much more predictive information the neural network *could include theoretically* in each layer, compared to how much it *actually includes*.

To be specific, from one representation *h*^(*l*)^ at layer *l* to another *h*^(*l*+1)^ at layer *l* + 1, spatial aggregation is the direct concatenation of all the neighboring representations

(5) $${\hat{h}}_{i}^{\left(l+1\right)}=\mathrm{concatenate}(h_{i+\delta }^{\left(l\right)},\;\mathrm{for all}\;\delta )\in R^{N_{l}\cdot |\{\delta \}|}$$

where *i* represents the voxel location, *N*_*l*_ is the number of channels in layer *l*, *δ* is the neighborhood, with |{*δ*}| usually being 27. We call ${\hat{h}}^{\left(l+1\right)}$ *pre-convolution representations*.

Information synthesis then is the application of convolution, batch normalization, and activation functions on ${\hat{h}}^{\left(l+1\right)}$ to generate *h*^(*l*+1)^

(6) $$h_{i}^{\left(l+1\right)}=\mathrm{ReLU}(BN(W\cdot {\hat{h}}_{i}^{\left(l+1\right)}))\in R^{N_{l+1}}$$

where *W* is the weights of the convolution. We call *h*^(*l*+1)^ *post-convolution representations*.

Note that with this breakdown, we will need to train two random forest models, *M*^(*l*−)^ and *M*^(*l*+)^, for pre/post-convolution representations at each layer in the layer embedding analysis.

## IV. Model Output Calibration

It has been documented that deep neural networks trained for classification tasks often experience poor *probability calibration* [17], which means the output score does not have a true probabilistic meaning. For example, a 0.95 score does not really mean the voxel has a 95% chance of being an anomaly. In other words, the output score can carry little meaning in its absolute magnitude, functioning instead more as a binary vote, due to the overfitting of model in these data points. This can be problematic because a poorly calibrated output makes interpretation difficult when losing its probabilistic meaning. In a different perspective, a true probabilistic output in a classification problem also means a more reliable uncertainty estimation. In this case, the uncertainty is directly reflected by the model’s output magnitude (e.g. a 0.65 score means the model is 65% certain about its classification). Here, we introduce the need to *calibrate* the model output, which is to adjust the magnitude of the output score so that it reflects a true probabilistic meaning, and thus giving a better uncertainty estimation.

In this section, we are looking to use our proposed layer embedding analysis method, the deep random forest, for improving the baseline model in terms of a better confidence calibration and thus a more reliable probability calibration, and a better classification performance. To the best of our knowledge, this is the first work to use inner layer interpretability results to improve the overall neural network model’s results.

### A. Baseline II: Monte Carlo Dropout

Monte Carlo (MC) dropout [27] is one of the techniques employed in neural networks for uncertainty estimation. With dropout enabled during the testing time, the model generates a different output in every forward pass for the same input, approximating Bayesian inference in deep Gaussian processing. By averaging these multiple outputs, it is a computationally efficient way of increasing model output robustness and estimating model uncertainty. To implement this, we adopt the exact same architecture as in our baseline FCN model (Fig. 2), except that after every major layer there will be a 3D dropout layer.

### B. Baseline III: Ensemble of Networks

Another way to find a more calibrated and also more robust output is to use an ensemble of networks [29]. It simply averages predictions over multiple models trained on the same training set data. To implement, we initialize and train each sub-model with different random seeds. During test time, we average all model outputs to generate a calibrated output. However, such a method will require much more computational resources.

### C. Baseline IV: Platt Linear Scaling

It is also possible to calibrate the model output directly through post-processing [17]. Using the Platt scaling method [18], we can also optimize

(7) $$\sigma_{\mathrm{direct}}^{\prime }=\mathrm{softmax}(a\cdot z+b)$$

where parameter *a* and *b* is found through the validation set, *z* is the output logits before the *softmax* layer. Though demonstrated to have good calibration potential, this linear scaling method does not change the relative ordering of the output scores. For example, for every two predicted values on data point *i* and *j*, *σ_i_* ≥ *σ_j_*, the linearly scaled outputs will yield $\sigma_{i}^{\prime }\geq \sigma_{j}^{\prime }$. As a result, this linear mapping is not able to fundamentally change the model’s classification performance in terms of AUC and DSC, which is computed based on such relative ordering of the output scores.

### D. Proposed Method: Layer Embedding Guided Calibration

**Fig. 5.**
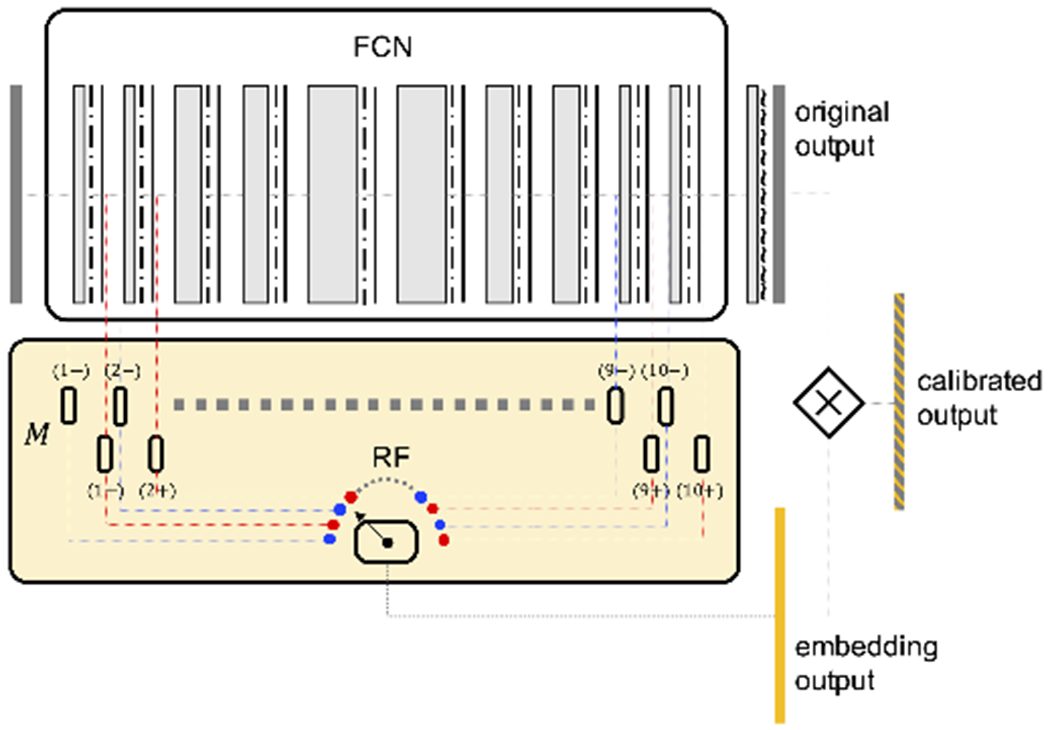
Overall pipeline for model output calibration using layer embedding outputs to improve probability calibration and classification. Inside the yellow RF block, blue/red colors symbolize pre/post-convolution representations explained in section III-C, small blocks are random forest models *M*’s for pre/post-convolution representations at each layer (1−), (1+), (2−), (2+), …, (10−), (10+).

Instead of a simple linear scaling of the FCN output, we hypothesize that the information we have in the intermediate layers should be useful in guiding the output calibration for improved performance. Since we know the neural network tends to solve easier problems first and more difficult ones later [6], confidence in intermediate layers could be a good indicator of task difficulties, thus be helpful in tuning model confidence in the final output. Therefore, we propose to weight the original FCN output *σ* with some function of the intermediate layers’ embedding output *σ*^(*l*)^ in order to give an estimation of how reliable this output is, thus improving both probability calibration and classification. This is also along the same thread of thinking with efforts using the deep k-nearest neighbors method for improving robustness against adversarial attacks, where consistency of intermediate layer predictions in the network is used to weigh the reliability of the ultimate output prediction. To be specific, we construct the calibrated output *σ*′ as a function of both the original model output *σ* and intermediate layer embedding output *σ*^(*l*)^

(8) $$\sigma^{\prime }=\sigma^{\prime }(\sigma ,\sigma^{\left(l\right)})=a\cdot \sigma^{\left(l\right)}\cdot \sigma +b$$

where parameter *a, b* and *l* can be learned from the validation set. In this study, we simply let *a* = 1 and *b* = 0 for a quick run, and only *l* is decided through the validation set, in order to reduce the number of parameters. Overall, we have this pipeline illustrated in Fig. 5. Note that in this illustration there are two models for each layer instead of just one, using the breakdown of the network described in section III-C.

## V. Experiments and Results

### A. Data Description

Our main dataset consists of 20 sets of 3D multi-parameter MR images on patients with HCC, each of which consisted of three T1 weighted DCE images at three different time points: pre-contrast phase (before the contrast injection), arterial phase (20 seconds after the injection), and venous phase (70 seconds after the injection). All three images were mutually registered to the space of the arterial phase image, with an isotropic voxel resolution of 1*mm* × 1 *mm* × 1*mm*, image dimension at around 100 × 100 × 100 after cropping. The ground truth of voxel labeling was completed by two radiologists, indicating whether a voxel belongs to background, parenchyma (healthy liver tissue), viable (enhanced) tumor tissue, or necrosis (Fig. 1).

### B. Implementation

Architecture setup for the baseline model is detailed in Table I. The dilation convolutions and the channel sizes are designed to mimic the traditional auto-encoder or U-net [37] symmetric structure. The total parameter number is around 320k. The training process takes around 48 hours to finish in 1000 epochs. We use a customized cross-entropy-based loss function and an Adam optimizer [38] for training. The model is trained on 44 × 44 × 44 patches. The augmentation includes random image contrast alteration, randomly free-form deforming the image, and adding random Gaussian noise. For each random forest model, we select the number of trees to be 20, with other hyper-parameters set to standard values in the python *scikit-learn* library. The training of all random forest models takes around 2 hours to finish. Five fold cross validation is used for evaluating the algorithm, splitting the dataset using a 60/20/20 ratio in each fold. All quantitative results in this study are calculated by aggregating predicted results from all folds. The baseline segmentation network is trained for a three class classification problem with background information given. During the layer embeddings analysis and calibration step, we focus only on the binary anomalies v.s. non-anomalies problem for ease of visualization and quantification.

### C. Layer Embedding Analysis

**Fig. 6.**
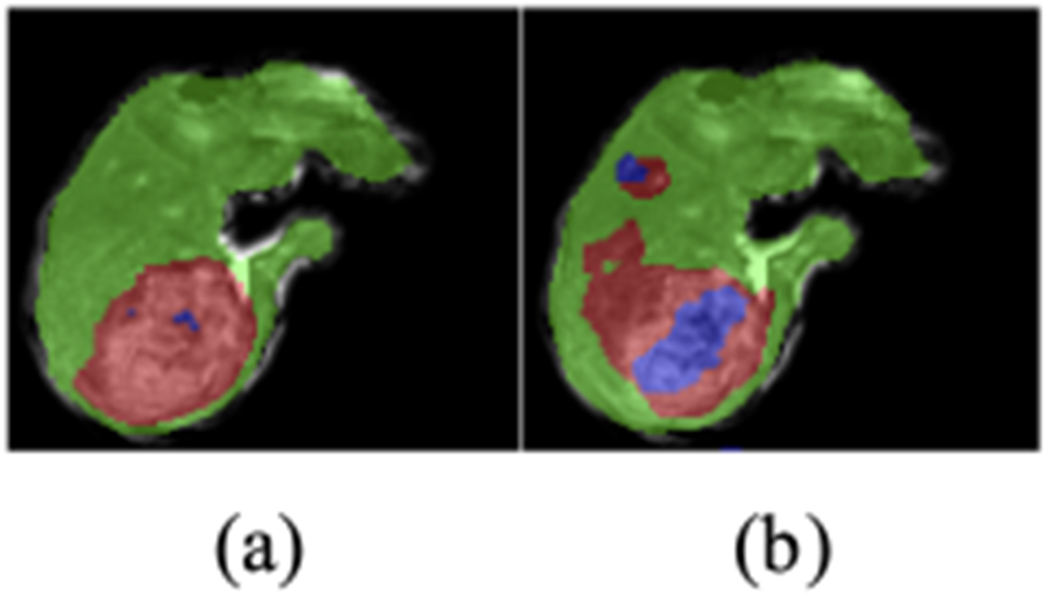
Baseline segmentation result, where green represents healthy liver tissue, red viable tumor tissue, blue necrotic tissue. (a) Ground truth; (b) Segmentation result from the original FCN.

Fig. 6 gives a demonstration of the baseline segmentation result. We can see that the model has a false positive detection on the top left corner, and a spill-over on the major anomaly.

**Fig. 7.**
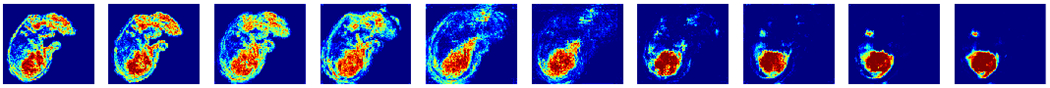
An illustration the progression of layer embedding output for anomalies using the deep random forest method. The color in the heatmap represents the likelihood of being an anomaly tissue, ranging from 0 to 1. From left to right are post-convolution representations in the hidden layers (1+, 2+, …, 10+).

Given this, what we are interested in then is how the network makes these predictions. So we look into the layer embedding outputs using our proposed deep random forest method (Fig. 7). Note that in this case, to simplify the problem, we are only looking at the binary classification problem of anomalies or non-anomalies (i.e. the output at each layer is one of these two binary classes). We can see intuitively that the network begins with a rough classification heat map that resembles the input image texture, and gradually refines to a mass mask through layers of spatial information aggregation and information synthesis operation. This behavior coincides with our understanding that the network is to extract lower level image features in earlier layers and high level content features in later layers. In this specific demonstration, we can also trace how the decision evolves across different layers in predicting a false positive anomaly. We can see that this false positive detection starts forming at layer 8 and eventually becomes a mass. Detailed quantification results using this will be explained in the next section.

**Fig. 8.**
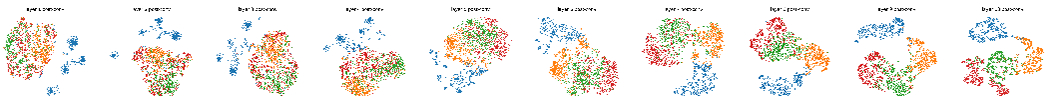
An illustration the progression of layer embedding morphology using t-SNE. Color blue represents background, orange healthy liver, green viable tumor, and red necrosis. From left to right are post-convolution representations in the hidden layers (1+, 2+, …, 10+).

We also show a demonstration of layer embedding morphology analysis using t-SNE (Fig. 8) for comparison. We can qualitatively observe that the embeddings separate different classes of data more and more as the layers go deeper. However, we are not able to quantitatively measure how well they separate, nor to trace how a specific sample (e.g. a false positive) progress throughout the layers.

### D. Model Calibration

**Fig. 9.**
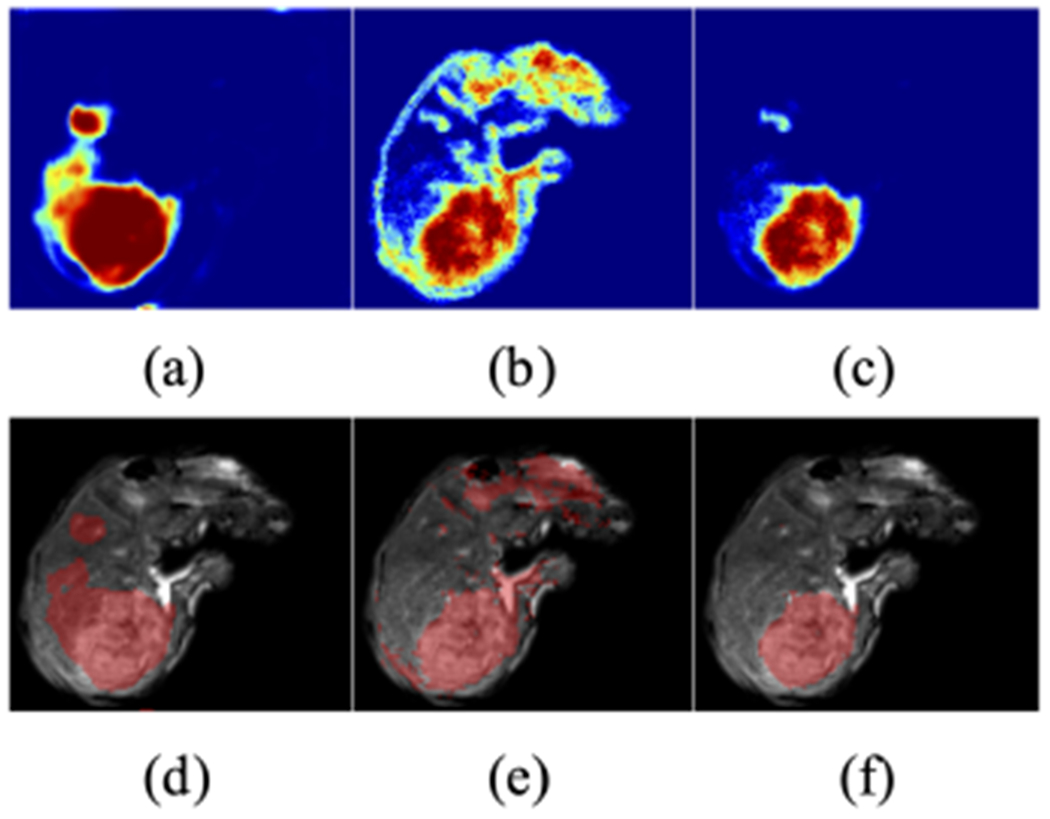
Demonstration of the calibration process. Anomaly probability map from (a) the original FCN *σ*; (b) pre-convolution representation embedding output *σ*^(2−)^; (c) our calibrated output *σ*′ that uses it. The color in the heatmap represents the likelihood ranging from 0 to 1. (d)-(f) Segmentation masks generated from (a)-(c). Note especially how false positives are de-emphasized in the calibrated output.

**Fig. 10.**
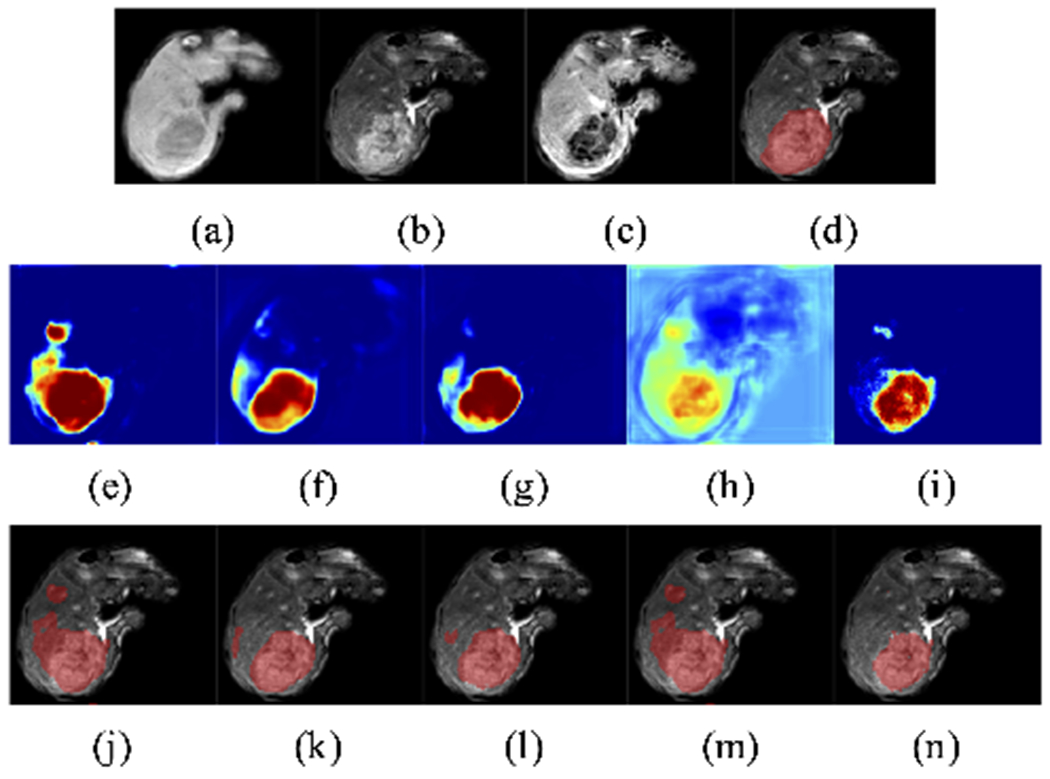
Demonstration of segmentation by different methods. (a)-(d) Three phases of MR images and ground truth segmentation for the anomaly. Probability output of (e) the original FCN; (f) MC dropout network; (g) the ensemble network; (h) Platt linear scaling; (i) the calibrated model output using pre-convolution representation embedding output at layer **2**. All colormaps are identical, ranging from 0 to 1. (j)-(n) Segmentation masks generated from (e)-(i).

**Fig. 11.**
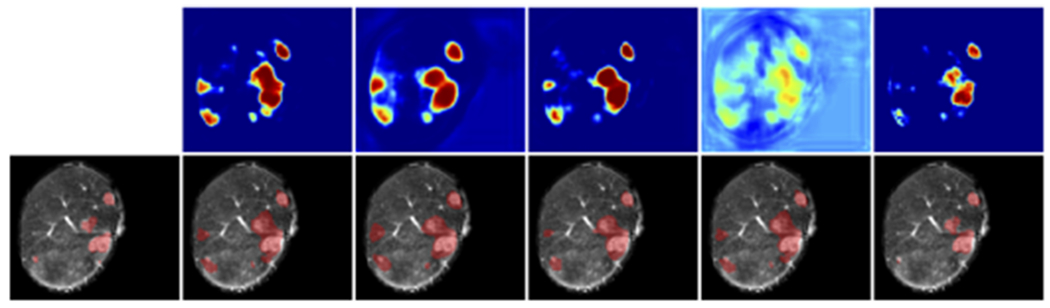
A second demonstration of segmentations arranged similarly to Fig. 10. From left to right: ground truth, original FCN, MC dropout, ensemble, Platt scaling, proposed calibration. The top row are the probability maps with identical colormaps, bottom row segmentation masks.

For a qualitative understanding, we present a demonstration (Fig. 9) of how the proposed calibration is carried out, and a demonstration (Fig. 10 and 11) of the anomaly classification map comparing our proposed calibration method and the four baseline methods: the original FCN model output (section II), MC dropout (section IV-A), ensemble of networks (section IV-B), and Platt linear scaling (section IV-C).

We next comprehensively investigate the performance of the embedding output at different layers, for both pre/post-convolution representations. Afterwards, calibrated model outputs using these embedding outputs, which is what we are ultimately interested in, are calculated. All of these are compared to the four baselines. We first perform such analysis in the validation set (first row in Fig. 13) to investigate the effect of *l* on model performance using calibration as in (8), and in the test set to show that this pattern is consistent with that in the validation set (second row in Fig. 13).

The performance is quantitatively measured in two aspects: *probability calibration* and *classification*. For probability calibration performance, we adopt the reliability diagram plot [39] and design an *average calibration error* (ACE) metric, defined as below. For classification performance, we use the conventional metrics AUC and DSC as mentioned previously.

#### 1) Probability Calibration Performance:

##### a) Reliability Diagram:

This graph plots the actual prediction accuracy against the output score, or so-called confidence. The accuracy is estimated empirically using bins of the data, $B_{m}=(\frac{m-1}{M},\frac{m}{M}]$

(9) $$\mathrm{accuracy}(B_{m})=\frac{\sum_{\sigma_{i}\in B_{m}}1({\hat{y}}_{i}=y_{i})}{\sum_{\sigma_{i}\in B_{m}}1}$$

so the plot will appear as a bin plot. Ideally, if the output score (confidence) reflects perfectly its true accuracy or certainty, the plot should mimic a diagonal line. A large deviation means a poor calibration and an inferior uncertainty estimation.

**Fig. 12.**
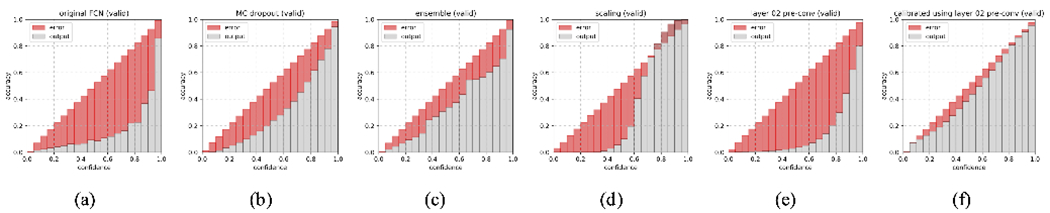
Reliability diagrams. The orange bars are the true accuracy for voxels at a certain predicted score (confidence), estimated using bins of data, blue bars calibration error. An ideal reliability diagram should have orange bars approximating a diagonal line, meaning the model output reflects a true probabilistic meaning, and a minimal area of blue bars. (a) Original FCN model; (b) MC dropout; (c) ensemble of networks; (d) Platt linear scaling; (e) pre-convolution embedding output at layer **2**; (f) our calibrated output that uses it.

Fig. 12 shows the reliability diagrams for the four baseline methods, one of the layer embedding outputs, and the calibrated model output that uses it. We can see that the original model output and the layer embedding output exhibit the least reliable probability calibration, whereas our calibrated model performs the best.

##### b) Average Calibration Error (ACE):

This metric is a quantification of the deviation from the ideal shown in the reliability diagram, i.e. the calibration error. We define it to be

(10) $$\mathrm{ACE}=\frac{1}{M}\overset{M}{\underset{m=1}{\sum }}\left|\mathrm{accuracy}(B_{m})-\mathrm{confidence}(B_{m}\right)|$$

which captures the model’s inherent calibration capability regardless of the class distribution in the input data.

**Fig. 13.**
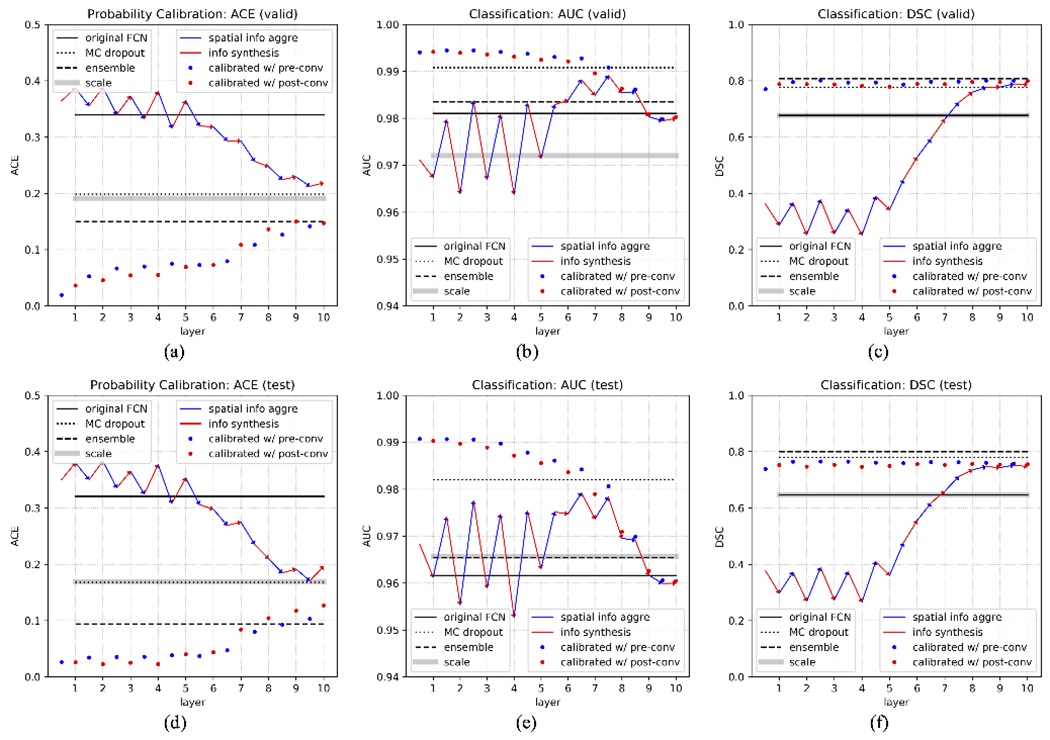
Comprehensive evaluation of performance in probability calibration, using Average Calibration Error (ACE), and classification, using Area Under Curve (AUC) and Dice Similarity Coefficient (DSC). Blue/red solid lines are the layer embedding output at pre/post-convolution representations. Blue/red dots are our calibrated output using pre/post-convolution representations at respective layers. Blue arrows represent the spatial information aggregation, generating pre-convolution representations. Red arrows represent the information synthesis, generating post-convolution representations. The black solid line represents the original FCN output, black dotted line MC dropout, black long dash line ensemble network, thick gray line Platt linear scaling. (a) ACE (b) AUC (c) DSC in the validation set to investigate the effect of *l* on model performance using calibration as in (8), and (d)-(f) in the test set to show that such performance from the calibrated model can be generalized to the test set. Note that our calibrated output using early layers generates the lowest ACE in (a) and (d), highest AUC in (b) and (e), and comparable DSC in (c) and (f). Detailed discussions in section V-D.

With this, we calculate the ACE of the embedding output from different layers, at both pre/post-convolution representations, as well as the calibrated model outputs using these embedding outputs (Fig. 13).

In this, we first see that when calibrated using *early layer* embedding outputs, our proposed calibration method has the lowest error, meaning it generates the most reliable probability calibration. At the same time, we see that the original FCN output has the highest (worst) ACE, whereas ensemble, MC dropout, and Platt linear scaling can improve the calibration considerably. Second, as the layers progress, layer embedding ACE drops, suggesting a better probability calibration. The gap between the last hidden layer and the original FCN output suggests a sub-optimal bias term in the last convolution. Third, calibration error increases when the model is calibrated using later layers, because the embedding will become more and more similar to the FCN output embedding as it progresses. More observations in terms of the pattern of the layer embedding output progression will be discussed in the next section.

#### 2) Classification Performance:

In evaluating the classification performance, we compute AUC and DSC for both pre/post-convolution representations at each layer embedding output, and also the calibrated model outputs that use these (Fig. 13). All of these are again compared to the four baselines.

Here, we make several observations. First, in terms of AUC, we again see that our proposed calibration method has the best performance when calibrated using early layer embedding outputs. In DSC, it is slightly lower than the two other baselines. This can partly be caused by the arbitrary values of parameter *a* and *b* in (8). Since the calculation of DSC depends on a threshold, after our calibration, the ideal threshold may no longer be 0.5, hence compromises the DSC score. For AUC, which measures the overall classification performance regardless of threshold selection, our method stands out to be the best.

Second, we observe that the embedding output AUC eventually converges to the original FCN output AUC, suggesting the layer embedding becomes more and more similar to the output embedding as the layer goes deeper, which fits our expectation. However, in the DSC curve, we notice the gap between the last layer of embedding output and the original FCN output, which is not present in the AUC curve. We think this could be caused by a sub-optimal bias term between the last hidden layer and the output layer. This sub-optimal bias effectively shifts the threshold for classification, thus affecting the DSC score, whereas AUC remains unaffected. For this reason, we in general see AUC as a more robust measurement of classification performance compared to DSC because of its independence from threshold selection.

Third, when using our proposed method to calibrate the FCN output using layer embedding information, we find that the performance generally declines when combined with information from later layers. This is because as the embeddings progress through layers, it becomes more and more similar to the FCN final output embedding, which means it will be more and more likely to suffer from the same overfitting error as that in the original output. Therefore, in this situation, combining those two will only see limited improvement.

Last but not least, we find some interesting behaviors that, to the best of our knowledge, has not yet been documented in other studies. In particular, we observe this zigzag pattern of layer embedding performance in the earlier layers, instead of increasing monotonically as we expect. In both AUC and DSC, the spatial information aggregation operation consistently increases the performance, which is to be expected since aggregating more spatial information will help prediction. However, the information synthesis operation does not always preserve all the available spatial information, generating a “worse” classification performance than that at the prev-convolution representations. We put a quotation mark on our description “worse” because we are not sure whether this is a desirable pattern of the neural network model. It could be a mechanism for the network to add regularization and prevent over-fitting, reserving the function of drastic data cluster shifting to later layers where higher level features are extracted. Or it could suggest that the model is not optimally trained, which leads us to wonder what would be the “ideal” pattern of such embedding outputs, and what would make that happen (network architecture, loss function design, etc).

### E. Generalization to a Public Dataset

**Fig. 14.**
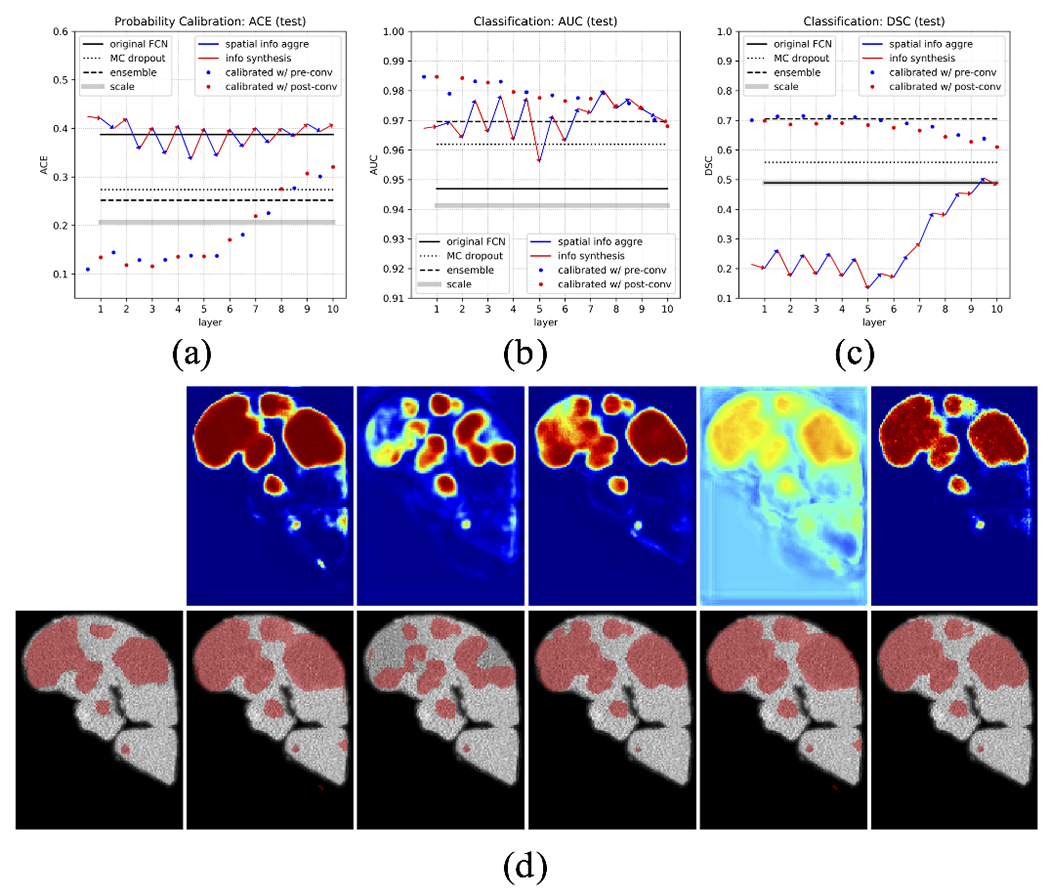
Comprehensive evaluation of performance in probability calibration (ACE) and classification (AUC and DSC) on the *LiTS dataset*. (a) ACE; (b) AUC; (c) DSC in the test set with the same legends as those in Fig. 13. (d) A demonstration of segmentations with the same arrangement as those in Fig. 11.

A supplementary dataset from the public domain, the liver tumor segmentation (LiTS) competition [40] (Fig. 14), is used to demonstrate the generalizability of our proposed method. This dataset consists of 131 3D CT images with segmentation masks on liver and anomalies.

For a quick test, we adopt the exact same architecture for training in this new dataset without hyper-parameter tuning. We show the demonstration of segmentation results for different methods and a comprehensive quantitative comparison in Fig. 14 in test set. The results are consistent with those in our MRI dataset, suggesting good generalizability of our proposed method.

## VI. Discussions

For most of the modern architectures that incorporate the attention mechanism [41], [42] or the residual connections [43]–[45], though architecturally much more sophisticated than the FCN network, they can often be broken down into building blocks that are more manageable for applying our methodology. Here, we offer discussions and some preliminary results in this direction.

### A. Application in Other Layers

In this study, our choice of FCN model only incorporates layers such as dilated convolution, batch normalization, and ReLU activation (section II). The rationale is that such minimal design can make convenient subsequent analysis and implementation. A look into how our calibration method can be implemented in other types of layers is definitely worthwhile, especially, in pooling layers and other activation functions.

Pooling layers such as max-pooling and average-pooling are ubiquitous in many modern networks, whose functionality has been studied empirically and theoretically [46]. However, pooling/upsampling layers will change the dimension of the layer embedding outputs, meaning they cannot be used for calibration directly. To solve this, one can resize the layer embedding outputs to the same dimension as the original model output.

Investigation on the impact of activation functions on model convergence and model embedding progression will also be interesting. In our study, it is hypothesized that the oscillating performance patterns in early layer embeddings could be a result of information compression, which is partly attributed to the cutoff in ReLU activation functions. Models with other types of activation, such as leaky ReLU [47] or sigmoid, may present a different behavior.

### B. Application in Architectures with Skip Connections

Using intermediate layer embedding outputs for model calibration in networks with skip connections will require modifications of our method. The addition of skip connections means the model can no longer process the signal in a Markovian manner. In fact, it has been hypothesized that skip connections can dramatically change how the model converges and the functionality of its layers [48], [49]. Therefore our proposal of using only *one* intermediate layer to calibrate the output may no longer be an optimal solution (Fig. 5).

More sophisticated methods are possible in these more advanced architectures. For example, one could use the variance of all the layer embedding outputs for output calibration. Alternatively, one could also select layers with branches for calibration. Whichever approach it is, the optimal solution will require a much more comprehensive study on a case-by-case basis. This is beyond the scope of our current study.

**Fig. 15.**
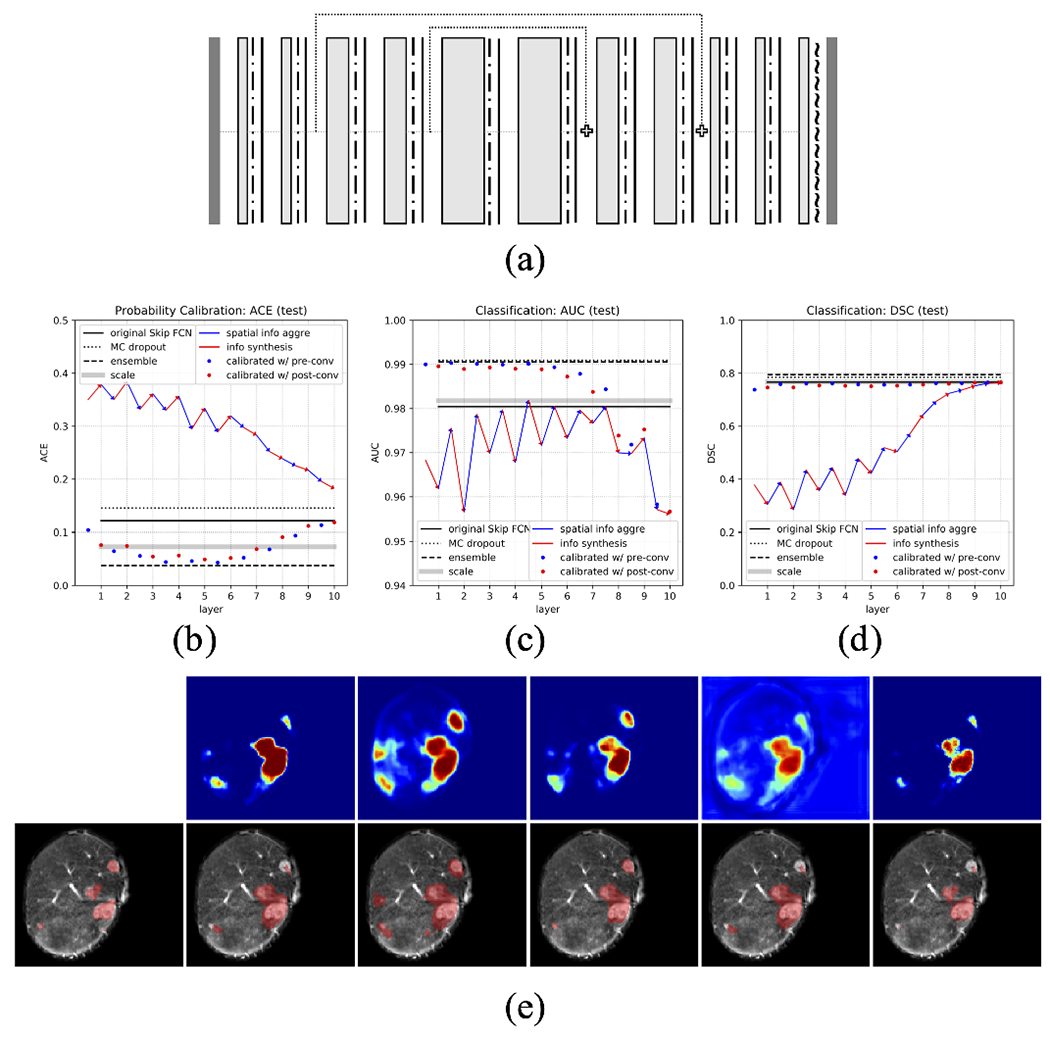
Comprehensive evaluation of performance in probability calibration (ACE) and classification (AUC and DSC) on a different architecture: (a) *FCN with skip connections* with the same legends as those in Fig. 2. (b) ACE; (c) AUC; (d) DSC in the test set with the same legends as those in Fig. 13. (e) A demonstration of segmentations with the same arrangement as those in Fig. 11.

**Fig. 16.**
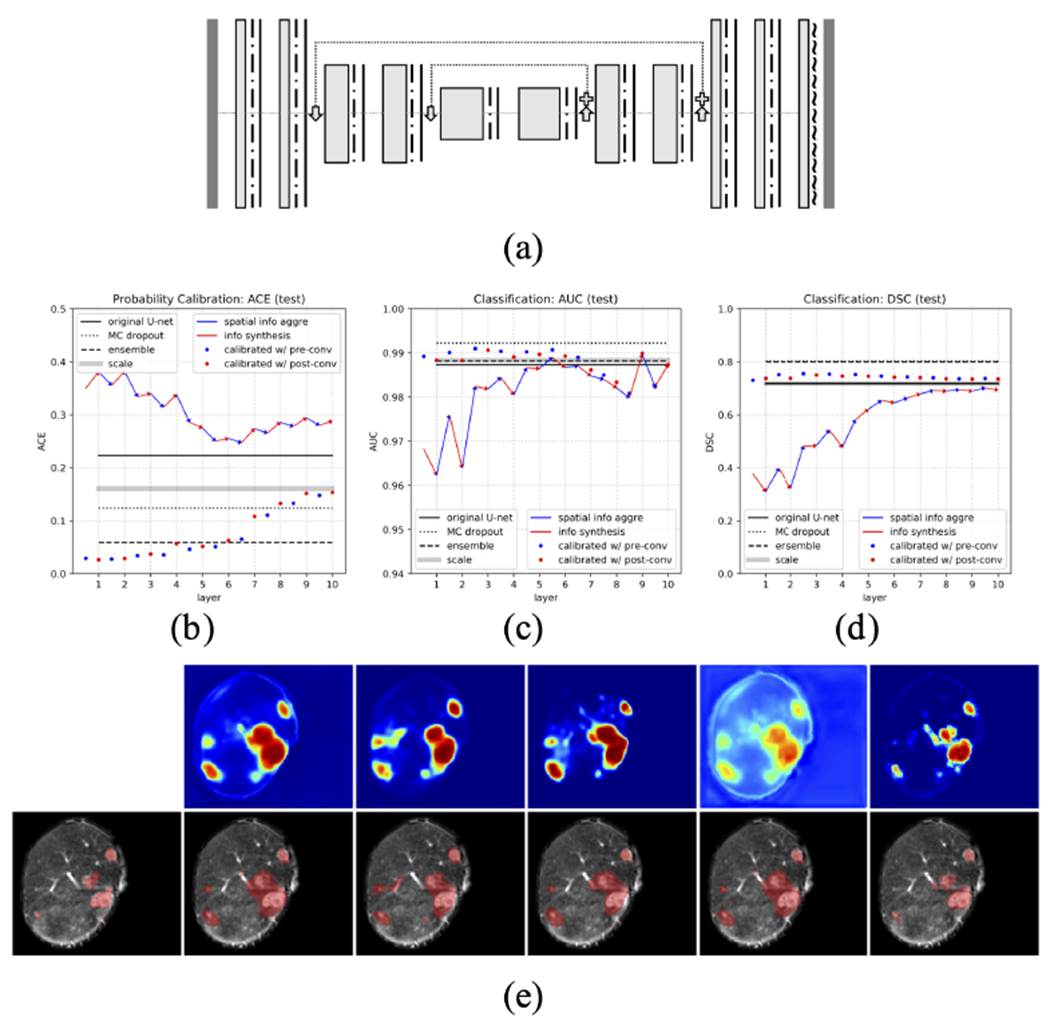
Comprehensive evaluation of performance in probability calibration (ACE) and classification (AUC and DSC) on a different architecture: (a) *U-net* with the same legends as those in Fig. 2. (b) ACE; (c) AUC; (d) DSC in the test set with the same legends as those in Fig. 13. (e) A demonstration of segmentations with the same arrangement as those in Fig. 11.

Nevertheless, we present here the results of implementing our one-layer calibration method on models with skip connections in order to show that the information in the intermediate layers can still be useful in improving confidence calibration and classification, and also to provide data and insights for future investigations. To be more specific, we test our probability calibration method in two other architectures that include max-pooling and skip connections in different fashions: a) FCN with skip connections (Fig. 15), and b) U-net [37] (Fig. 16). The new models share the same hyper-parameters as those in Table I except the addition of skip connections and max-pooling/upsampling layers in corresponding locations. Overall, we see that using some intermediate layer embedding information can improve probability calibration over the original model, with performance exceeding or on par with the best benchmark, and classification performance is improved in different scales. Interestingly, in FCN with skip connections, the best layers for calibration locate in the middle part of the model, suggesting an altering of layer functionality compared to the original FCN. However, in U-net, with the addition of max-pooling that perhaps has changed how information is processed, earlier layers again become the better option for calibration.

### c. Combination of Different Techniques

In this study, our layer embedding calibration method is presented as a stand-alone technique for model output calibration, compared against other benchmark techniques (MC dropout, ensemble, and linear scaling). However, it is worth noting that, just as with most of the deep learning modules we have seen in the past, all of these can be used in combination with others. For example, one could use Platt scaling on top of our layer embedding calibration method, or on MC dropout [31]. The effectiveness of such strategies is to be explored and not within the scope of our current study.

## VII. Conclusions

Overall, we highlight our findings in two parts. In the first part, we designed a method, the deep random forest, for inspecting the decision making process in a FCN. To be specific, we construct random forest models to approximate the data distribution on the embeddings at each layer, whose output is defined as the embedding output. In the task of liver tissue classification in our institutional MRI dataset, we can use this to observe how the network extracts and refines the segmentation boundary layer by layer, and to trace the decision progression toward its final prediction, especially when it comes to a false positive anomaly prediction. Such information is potentially useful in facilitating our understanding of the neural network, creating a more reliable and transparent decision making process.

In the second part, we show that this insight into layer embeddings can be used for improving model performance in probability calibration and classification. To do this, we use the embedding outputs in the hidden layers to calibrate the original FCN output, knowing that confidence in early embeddings can serve as a measure of task difficulty. We quantitatively show that this simple technique generates a good probability calibration in terms of ACE, and achieves a classification performance level that is better than other baseline methods in terms of AUC, and at a similar level in terms of DSC. This result on our institutional MRI dataset is consistent with that on a public CT dataset, suggesting our method’s generalizability.

By using a unique way of decomposing the FCN into a series of basic operations, namely, spatial information aggregation and information synthesis, we are also able to discover some interesting patterns that are counter-intuitive, which can potentially provide us insights for future design.

Our proposed method for analyzing layer embeddings can be helpful in investigating other questions that are important in the neural network community. An application in more advanced architectures, such as U-net or res-net, would be interesting, especially for analyzing how skip connections change the functionality of the layer embeddings and help classification.

## Figures and Tables

**TABLE I:** Architecture hyper-parameter setup used in the baseline FCN model.

layer	input	1-2	3-4	5-6	7-8	9-10	output
struct	-	ReLU(BN(conv(−)))					softmax(conv(−))
shape	-	3x3x3					1x1x1
chan	3	16	32	64	32	16	4
dilate	-	1	2	4	2	1	-
